# Subacute Histoplasmosis with Focal Involvement of the Epiglottis: Importance of Differential Diagnosis

**DOI:** 10.1155/2014/235975

**Published:** 2014-02-05

**Authors:** F. Ahumada, D. Pérez, M. de Górgolas, B. Álvarez, A. Ríos, A. Sánchez, JM. Villacampa

**Affiliations:** ^1^Department of Otolaryngology, Head Neck Surgery, Hospital Universitario Fundación Jiménez Díaz, Avenida de Reyes Católicos 2, 28040 Madrid, Spain; ^2^Department of Internal Medicine, Hospital Universitario Fundación Jiménez Díaz, Avenida de Reyes Católicos 2, 28040 Madrid, Spain; ^3^Department of Anatomic Pathology, Hospital Universitario Fundación Jiménez Díaz, Avenida de Reyes Católicos 2, 28040 Madrid, Spain

## Abstract

Histoplasmosis is an endemic mycosis of the Americas, Africa, and Asia. In Spain, it is the most common imported endemic mycosis appearing in the literature, and its incidence is on the rise. Proper differential diagnosis of the disease must be taken into consideration by otorhinolaryngologists, as the clinical manifestations of histoplasmosis may simulate more prevalent diseases such as cancer or tuberculosis. We present the case of a Spanish patient with focal involvement of the larynx and offer a review of the relevant literature.

## 1. Introduction

*Histoplasma capsulatum*, the etiologic agent of histoplasmosis, causes more infections in humans than any other endemic mycosis. As with other fungal diseases, initial exposure to the fungus occurs through inhalation. Cases of the disease have been described worldwide, although it is endemic to North America, Latin America, and particular regions of Africa and Asia. Over 80% of the people who live in the areas around the Ohio and Mississippi River Valleys present serological signs of infection [1].

Clinical manifestation is varied and may affect all the organs and tissues in the body. When the inoculum is small in size, most infections are asymptomatic. Immunodeficient patients and patients infected by large inoculums of fungal organisms may develop more severe or disseminated infections. Firm diagnosis is reached by isolating the fungus in special cultures, while alternate methods include searching for yeast forms in diseased tissues or detecting serum antibodies or specific antigens.

Treatment consists of intravenous amphotericin B antifungal therapy for 10 days in addition to oral itraconazole for 9 to 12 months.

It is important for ENT specialists to take into account the differential diagnosis of this illness, as the clinical manifestations of histoplasmosis in the oropharynx and larynx may resemble malignant neoplasia or tuberculosis [2].

## 2. Clinical Case

A 71-year-old male patient from Madrid, Spain, presented to our department with dysphonia that had been present for 2 months. He reported a 30-year history of smoking and had received a liver transplant 8 years before as a result of liver cirrhosis, for which he was undergoing chronic immunosuppressant treatment with mycophenolate mofetil.

The patient presented manifestations of dysphonia, dysphagia, odynophagia, and a sensation of autophony, together with fatigue, anorexia, and a loss of over 20 kg of body weight in the previous 6 months.

**Figure 1:**
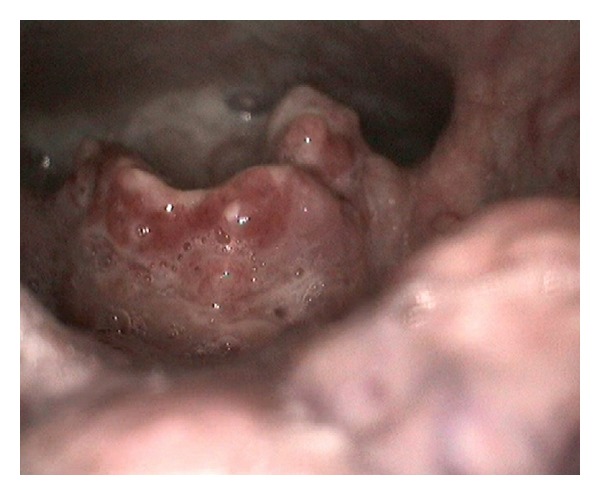
A fiber-optic laryngoscopy revealed a vegetative lesion on the lingual surface of the epiglottis.

**Figure 2:**
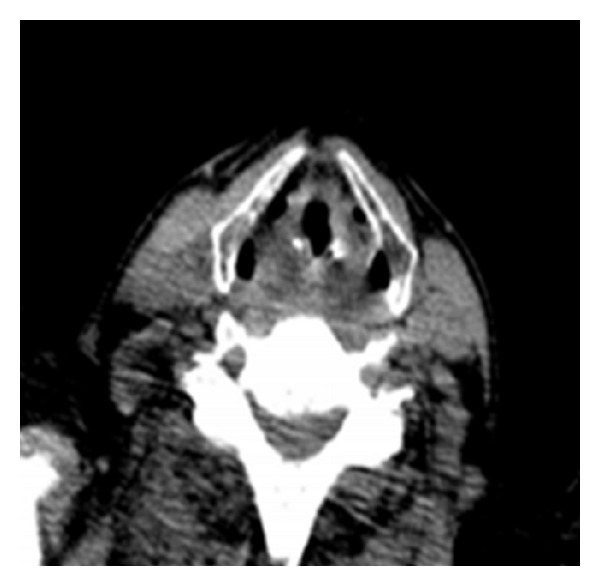
Computed tomography scan of the neck. The images obtained showed increased size of the soft tissue on the lingual surface of the epiglottis.

**Figure 3:**
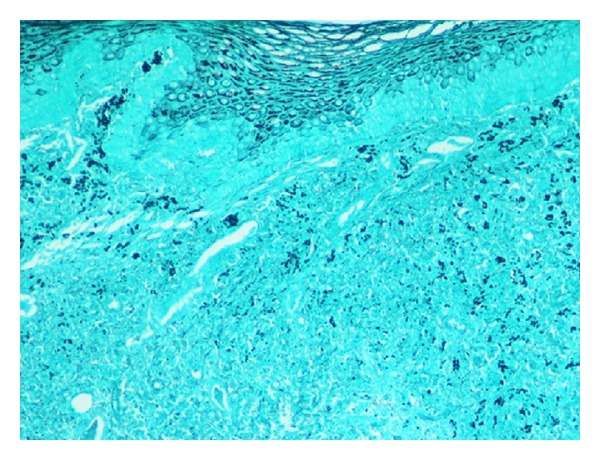
The biopsy revealed an ulcer base with fungus.

A fiber-optic laryngoscopy (Figure 1) revealed a vegetative lesion on the lingual surface of the epiglottis. Because involvement reached the epiglottic vallecula, a biopsy was taken during the examination. Computed tomography (CT) was performed with no contrast due to a previous history of allergy. The images obtained showed increased size of the soft tissue on the lingual surface of the epiglottis (Figure 2). The biopsy (additional histochemical analysis with Ziehl-Neelsen staining and Grocott staning) revealed an ulcer base with fungus (spores with some budding) (Figure 3). These findings were compatible with histoplasmosis.

In light of the findings, the patient was asked whether he had traveled to endemic areas; he reported having traveled to Central America in his youth while working for a maritime company.

We performed a bone marrow biopsy, bronchoalveolar lavage, blood cultures, CT of the head, chest, and abdomen, and adrenal function test. No evidence of additional histoplasmosis was found during this study of disease extension.

**Figure 4:**
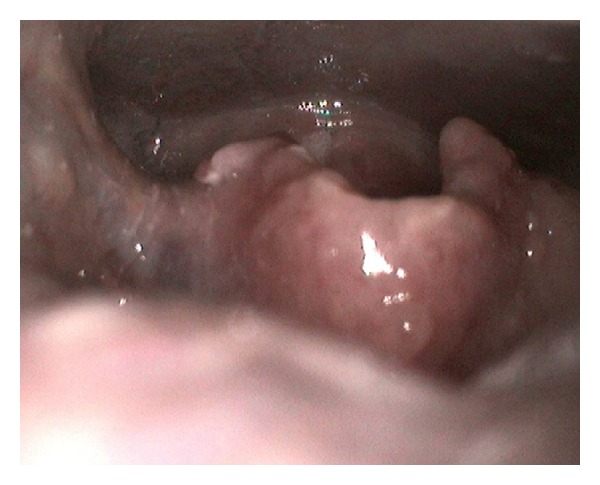
A study using fiber-optic laryngoscopy performed 4 months into the treatment revealed that the lesions had disappeared.

The patient was prescribed intravenous amphotericin B at a dose of 0.8 mg/kg/day for 10 days. At discharge, he continued receiving oral itraconazole (400 mg/day) prescribed for 12 months. Complete remission of the clinical symptoms was achieved during follow-up visits. Also, a study using fiber-optic laryngoscopy performed 4 months into the treatment revealed that the lesions had disappeared (Figure 4). At the time of writing (14 months into the treatment), no signs of recurrence have been observed.

## 3. Discussion

Histoplasmosis is the most common endemic mycosis seen worldwide, especially in the Americas, Africa, and Asia. The disease is not prevalent in Europe, although in Spain the incidence of the disease is now higher due to a number of factors such as an increase in immigration from endemic areas, increased travel to these areas, and a higher number of immunodeficient patients. Buitrago and Cuenca-Estrella report that, since the 1980s, 128 cases have been described in Spain; 59 of these individuals were travelers, 63 were immigrants, and 6 were patients originally from Spain. Of these 6 Spanish cases, 3 infections were related to the consumption of contaminated drugs, 2 were infected in laboratories, and 1 contracted the disease after receiving a solid-organ transplant [3].

The disease has a wide range of clinical manifestations, and though a vast majority of cases are asymptomatic, other infections lead to fatal illness. Acute infection with the *Histoplasma capsulatum* fungus presents clinically in less than 1% of patients with manifestations such as acute pulmonary histoplasmosis, chronic cavitary pulmonary histoplasmosis, mediastinal fibrosis, and pericarditis [4]. Progressive disseminated histoplasmosis presents mainly in immunodeficient patients (due to acquired immunodeficiency syndrome, use of immunosuppressants, receipt of solid-organ transplants, old age, etc.) [5]. In such cases, the disease tends to evolve from an acute form involving primarily the lungs and then spreads to other organs through the bloodstream [6]. Infection may also be subacute, exhibiting focal involvement of the adrenal organs, the gastrointestinal tract, the oropharyngeal mucosa, and the larynx. Subacute manifestations may present as ulcerations, ulcerated nodular lesions, granulomas, or verrucous lesions in the affected organs [7]. Although our patient had received an organ transplant and was undergoing immunosuppressant therapy, no disease manifestations were in evidence outside of the larynx, which leads us to the reasonable conclusion that the infection was not disseminated.

The disease may be diagnosed by culturing the fungus, with test results taking up to 6 weeks [4, 6]. An alternative method is a histopathological study to detect the intracellular yeast cells of *Histoplasma capsulatum* using Giemsa and Grocott staining, which allows the yeast cells to be distinguished from similar microscopic features [8].

Differential diagnosis is essential because the lesions and clinical characteristics of the disease may resemble those of neoplasms [9] or laryngeal tuberculosis, thus delaying appropriate therapy. A number of cases of laryngeal histoplasmosis have been described in which the infection was treated as disseminated tuberculosis for months [10], while, in other cases, the disease presentation has mimicked that of pharyngeal or laryngeal carcinoma [11].

The treatment of choice for histoplasmosis is intravenous amphotericin B administered for 7 to 10 days followed by oral itraconazole taken for between 9 and 12 months. The prognosis following this treatment is favorable [12–14].

## 4. Conclusion

It appears that the incidence of histoplasmosis is on the rise in Spain, and the involvement of the pharynx and larynx should be taken into consideration during the differential diagnosis of lesions affecting the upper airway and digestive tract so as to accurately rule out malignant neoplasms and tuberculosis, especially in immunodepressed patients, immigrants, and individuals who have traveled to endemic areas.
